# Medial Patellofemoral Ligament Reconstruction With Transverse Reefing (Tubing) of Medial Patellar Retinaculum and Biological Fixation

**DOI:** 10.1002/atn2.70327

**Published:** 2026-11-12

**Authors:** Hadi Makhmalbaf, Sepehr Hoseinzadeh Moghaddam, Abulfazl Vatankhah, Mohammad H. Ebrahimzadeh, Mahdieh Samei, Reza Ganji

**Affiliations:** ^1^ Orthopedics Research Center Ghaem Hospital, Mashhad University of Medical Sciences Mashhad Iran; ^2^ School of Medicine North Khorasan University of Medical Sciences Bojnurd Iran; ^3^ Natural Products and Medicinal Plants Research Center North Khorasan University of Medical Sciences Bojnurd Iran; ^4^ Department of Orthopedic Surgery School of Medicine, North Khorasan University of Medical Sciences Bojnurd Iran

## Abstract

The medial patellofemoral ligament (MPFL) is regarded as one of the main stabilizers of the knee. Its importance is underscored by the fact that in most cases of acute lateral patellar dislocation, rupture of the MPFL occurs. It is also a key factor in recurrent patellar dislocations. Therefore, MPFL reconstruction surgery is the current approach to managing such cases. Numerous techniques have been practiced by surgeons, with some being more prevalent than others. Although there is extensive literature comparing the efficacy and safety of different techniques, no clear consensus has been reached. Furthermore, recurrent postoperative complications, such as knee pain, have prompted surgeons to seek new and less invasive techniques. In this technical note, we describe an implant‐free MPFL reconstruction method leveraging the medial patellar retinaculum. Through horizontal suturing, a reinforced tubular reef is created to mimic the native MPFL and is secured via a subperiosteal femoral pouch with minimal invasiveness. This approach avoids bone tunnels, and it is expected to reduce fracture risk, eliminate donor‐site morbidity, and enhance reproducibility for surgeons.

## Graphical abstract

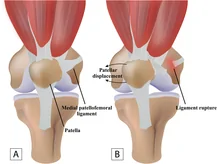

**VIDEO 1:** Preoperatively, a careful and individualized evaluation is conducted to identify concomitant anatomic or alignment abnormalities that may require correction. Then, under general anesthesia, a longitudinal medial parapatellar incision of approximately 6 cm is made. The medial femoral epicondyle and medial patellar border are palpated and marked. Adjacent to the medial epicondyle, the medial retinaculum is pulled upward, and suturing will start at the neck of the rolled‐up retinaculum. The first row of sutures continues toward the patella. By continuing the suturing, the reef began to form. Then, we palpate the medial border of the patella to make sure the sutures have reached the patella. The second row of sutures can be started by returning in the same direction toward the medial femoral epicondyle. In the second row, the sutures mirror the first row, covering unsutured gaps. The second row continues to about 2 cm from the medial femoral epicondyle. Then, a 1.5‐ to 2‐cm incision is made on either side of the medial end of the reef to release the constructed reef without compromising the sutures. As shown, the patella moves when the constructed reef is retracted. A 2‐cm longitudinal incision is made over the medial epicondyle to create a subperiosteal fascial pouch. The released construct, which is now mobile and able to be pulled medially together with the patella for about 2 cm, is pulled underneath the created pouch and fixed with sufficient tension by suturing it to the pouch and deep sutures into the adjacent periosteum. After sufficient fixation and verification of integrity, the surgery was concluded with the final surgical knot. The patella is checked for stability, and the knee is mobilized to check for optimal tension and to prevent overtensioning. Video content can be viewed at https://doi.org/10.1002/atn2.70327.

The medial patellofemoral ligament (MPFL) is the main static stabilizer of the patellofemoral joint, which primarily resists lateral displacement of the patella.^1^ Rupture of the MPFL is consistently reported in 95% to 100% of acute lateral patellar dislocations (Figure 1), showing its cardinal role in patellar stability and introducing the need for MPFL reconstruction.^2^ Additionally, in recurrent patellar instability, MPFL reconstruction has emerged as the standard surgical intervention if conservative treatment fails.^3^ Various surgical techniques have been proposed and adopted by surgeons to address the cause and treat instability with varying results; however, there remains considerable debate regarding optimal methods, graft choice, fixation sites, and tensioning strategies, thus reaching no consensus in this field.^4^, ^5^ Furthermore, despite various techniques being practiced, complications persist, with reported rates reaching up to 32.3%, primarily consisting of patellar fractures and anterior knee pain.^6^ These complications emphasize the ongoing need for less invasive, yet anatomically compatible techniques that provide reliable outcomes and minimize surgical morbidity. In our own surgical experience, we had long used the middle third of the quadriceps tendon for MPFL reconstruction, before adopting our current biological, implant‐free approach using the medial patellar retinaculum. We describe an MPFL reconstruction technique used since 2017, which reconstructs the MPFL by transverse reefing (tubing) of the medial patellar retinaculum, creating a construct that spans from the patella to the original point of insertion of the MPFL on the medial epicondyle.

**FIGURE 1:**
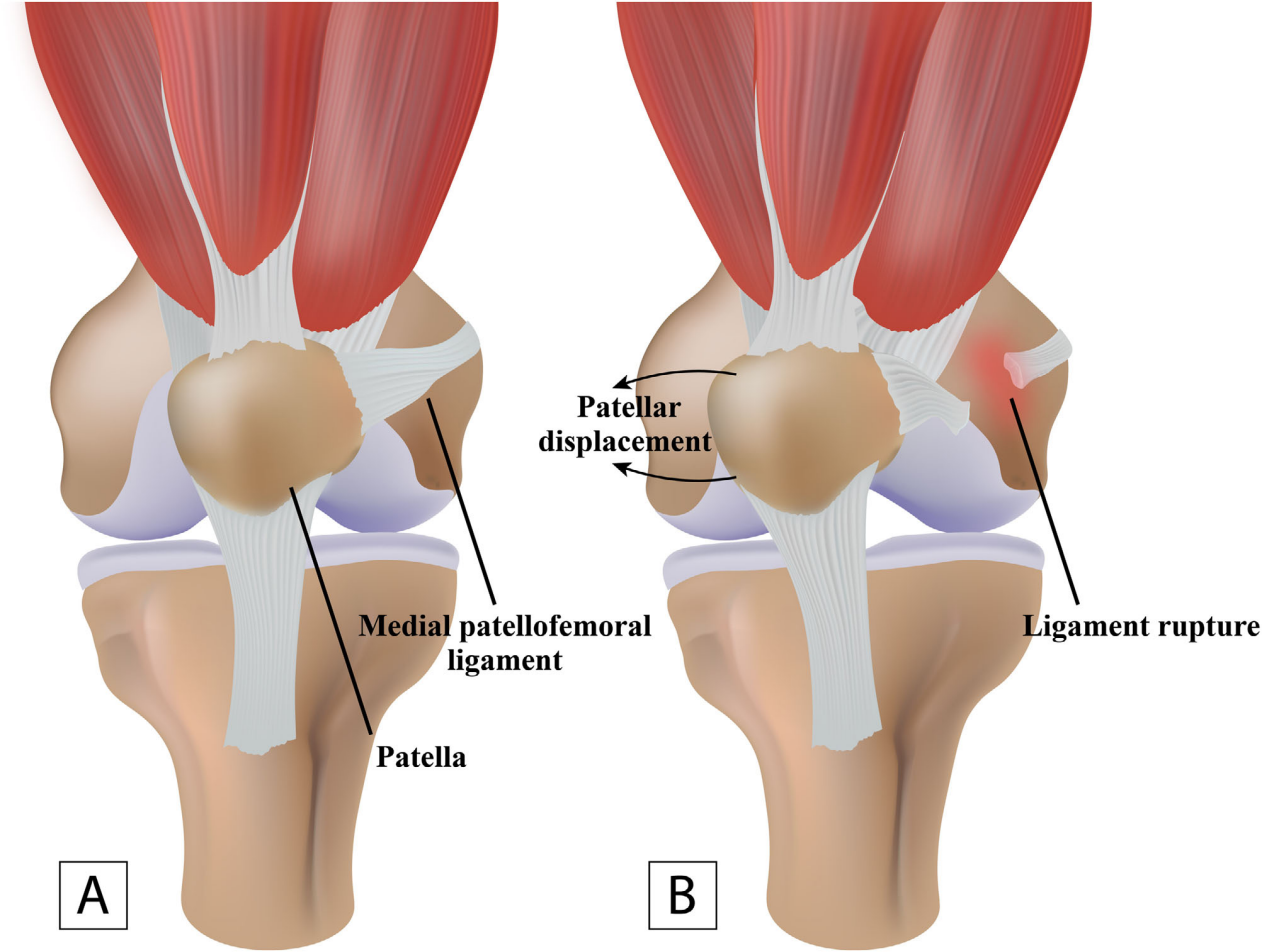
A schematic illustration of the knee joint and MPFL anatomy. (A) Normal anatomy of MPFL in a right knee, anterior view. (B) Lateral patellar displacement after MPFL rupture. (MPFL, medial patellofemoral ligament.)

## SURGICAL TECHNIQUE

### Patient Selection and Preoperative Planning

A careful and individualized plan is warranted to identify concomitant anatomic or alignment abnormalities that may require correction (Video 1). This evaluation includes assessment of the patient's overall alignment, patellar stability, apprehension test, valgus malalignment, patella alta, increased Q angle, J sign, and the presence of generalized ligamentous laxity or hypermobility. In adolescents with patellar instability, generalized joint laxity is frequently an underlying cause.^7^

Anteroposterior, lateral, and patellar radiographic views of both knees are mandatory to assess the position of the patella within the trochlear groove and to identify trochlear dysplasia and patella alta. Alignment radiographs are obtained when malalignment is suspected. Magnetic resonance imaging is also necessary to evaluate patellar position and MPFL integrity, detect concomitant chondral or osteochondral fractures, and identify loose bodies within the joint, as well as to assess the tibial tubercle‐trochlear groove distance and other differential diagnoses, such as anterior cruciate ligament tears. In patients with excessive valgus deformity, a distal femoral osteotomy may be required, either alone or in combination with MPFL reconstruction. In patients with an increased Q angle, medialization of the tibial tubercle may be indicated, and in cases of patella alta, distalization of the tibial tubercle may be performed concurrently with MPFL reconstruction. In patients with trochlear dysplasia (Dejour types B‐D), trochleoplasty is recommended. However, most of our patients have normal trochlear groove morphology, and our experience with this procedure is limited. Proximal and distal realignment techniques have been proposed to address patellar instability. Our technique represents a proximal realignment procedure that may be combined with a distal realignment when necessary to correct coexisting alignment abnormalities.

### Patient Positioning and Arthroscopic Assessment

Under general anesthesia with the patient in the supine position, an examination under anesthesia is performed to assess ligamentous laxity and patellar instability. We do not routinely perform arthroscopy unless intra‐articular pathology, such as osteochondral fractures or loose bodies, is present, which may require fixation or removal, respectively.

### Incision and Superficial Dissection

A longitudinal medial parapatellar incision of approximately 6 cm is made. Soft tissue over the retinaculum is retracted, and the medial retinaculum is exposed, extending from the patella to the medial epicondyle. The medial femoral epicondyle is palpated and marked.

### Reinforced Horizontal Reef Construction

We start reefing the medial patellar retinaculum, beginning adjacent to the medial epicondyle. It is pulled upward and sutured with No. 1 Vicryl (polyglactin 910) suture (Supa Medical Industries, Tehran, Iran) from underneath or at the neck of the rolled‐up retinaculum (Figure 2). The suturing continues toward the patella and then returns in the same direction to complete the sutures up to about 2 cm from the medial epicondyle (Figure 3). This technique provides good medial tether to deter the patella from dislocation and, at the same time, prevents overtightening of the reconstructed MPFL (Figure 4). Then, a 1.5‐ to 2‐cm incision is made on either side of the upward tubed construct, without compromising the sutures. The 2 ends of the sutures are preserved for later fixation (Figure 5).

**FIGURE 2:**
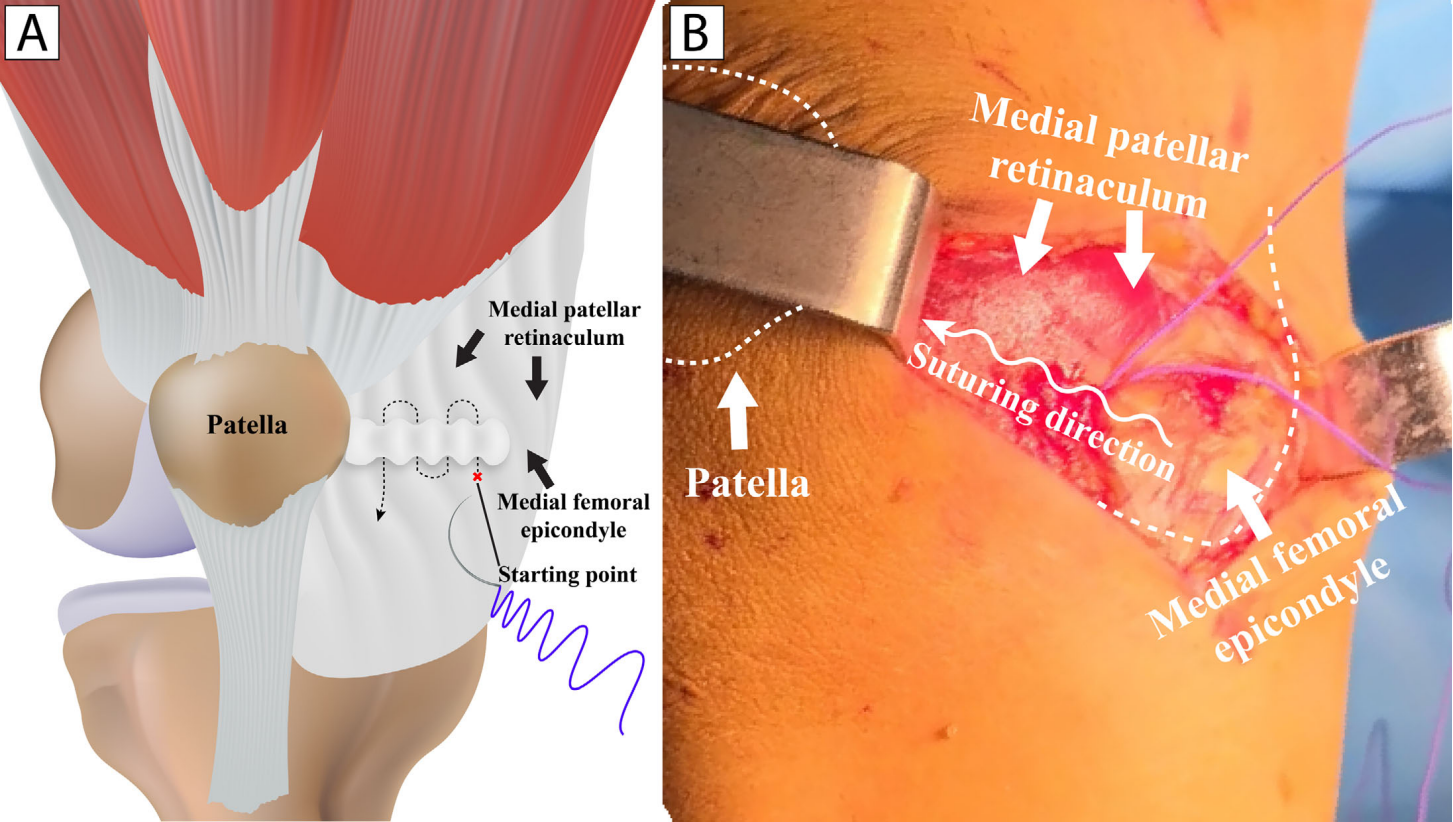
Starting the first row of sutures. (A) Schematic view showing the start of MPFL reconstruction. Adjacent to the medial femoral epicondyle (marked by a cross) after retraction of the medial patellar retinaculum, a continuous running suture is initiated at the neck of the rolled‐up retinaculum and directed toward the medial border of the patella (dashed arrow trajectory). This establishes the first pass of the retinacular reinforcement. (B) Corresponding intraoperative view showing the medial patellar retinaculum and suturing direction. The positions of the patella and medial femoral epicondyle are indicated by dashed lines. The patient is positioned supine with the knee in midflexion; this is the right knee. (MPFL, medial patellofemoral ligament.)

**FIGURE 3:**
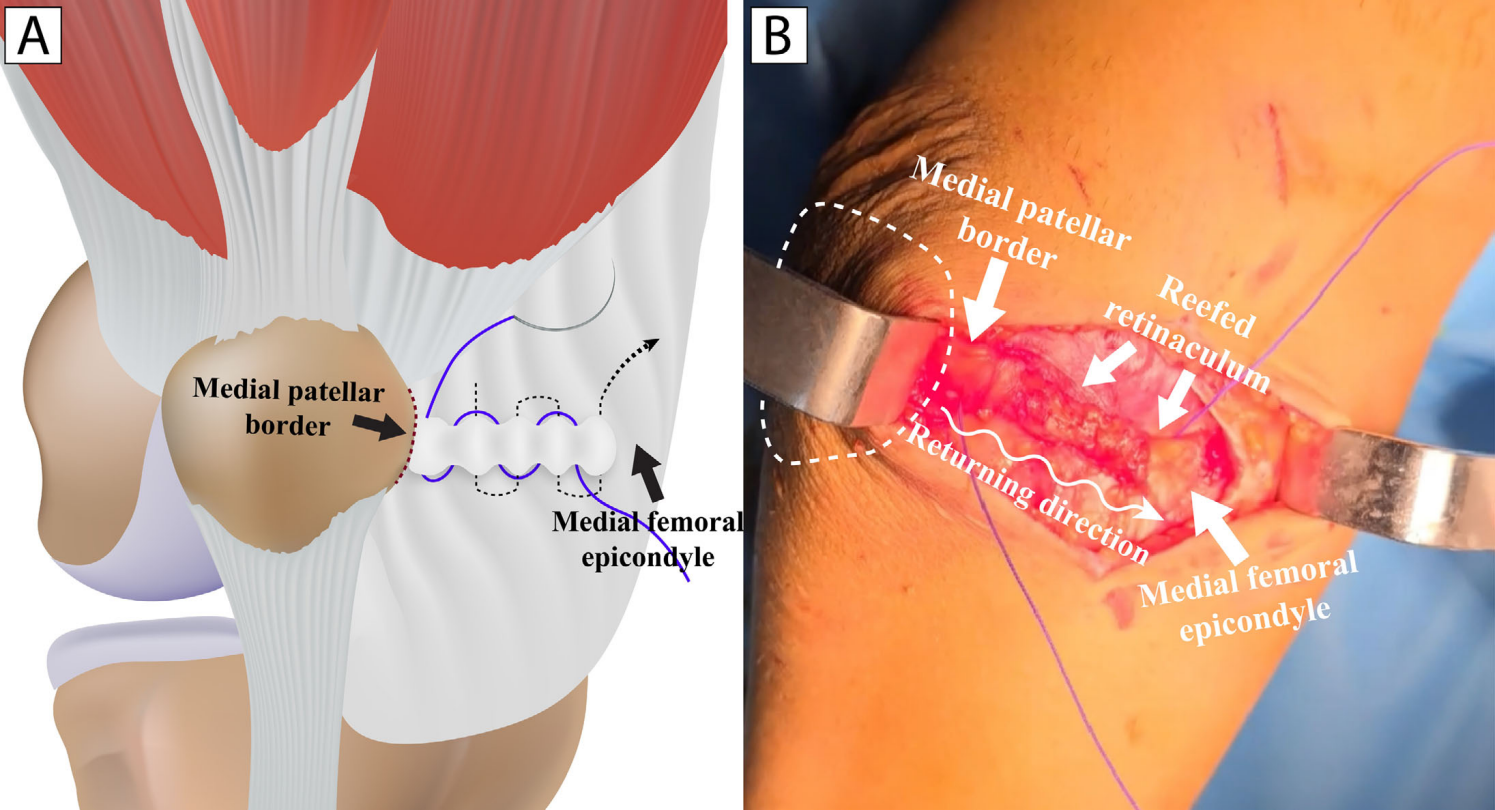
Second row of sutures. (A) Schematic illustration showing the suture reaching the medial border of the patella. The continuous suturing proceeds toward the medial femoral epicondyle (dashed arrow trajectory) to mirror the first row and fill the unsutured gaps. (B) Corresponding intraoperative view showing reef formation after the first row of sutures. The returning direction is indicated by an arrow. The positions of the patella and medial femoral epicondyle are marked by dashed lines. The patient is positioned supine with the knee in midflexion; this is the right knee.

**FIGURE 4:**
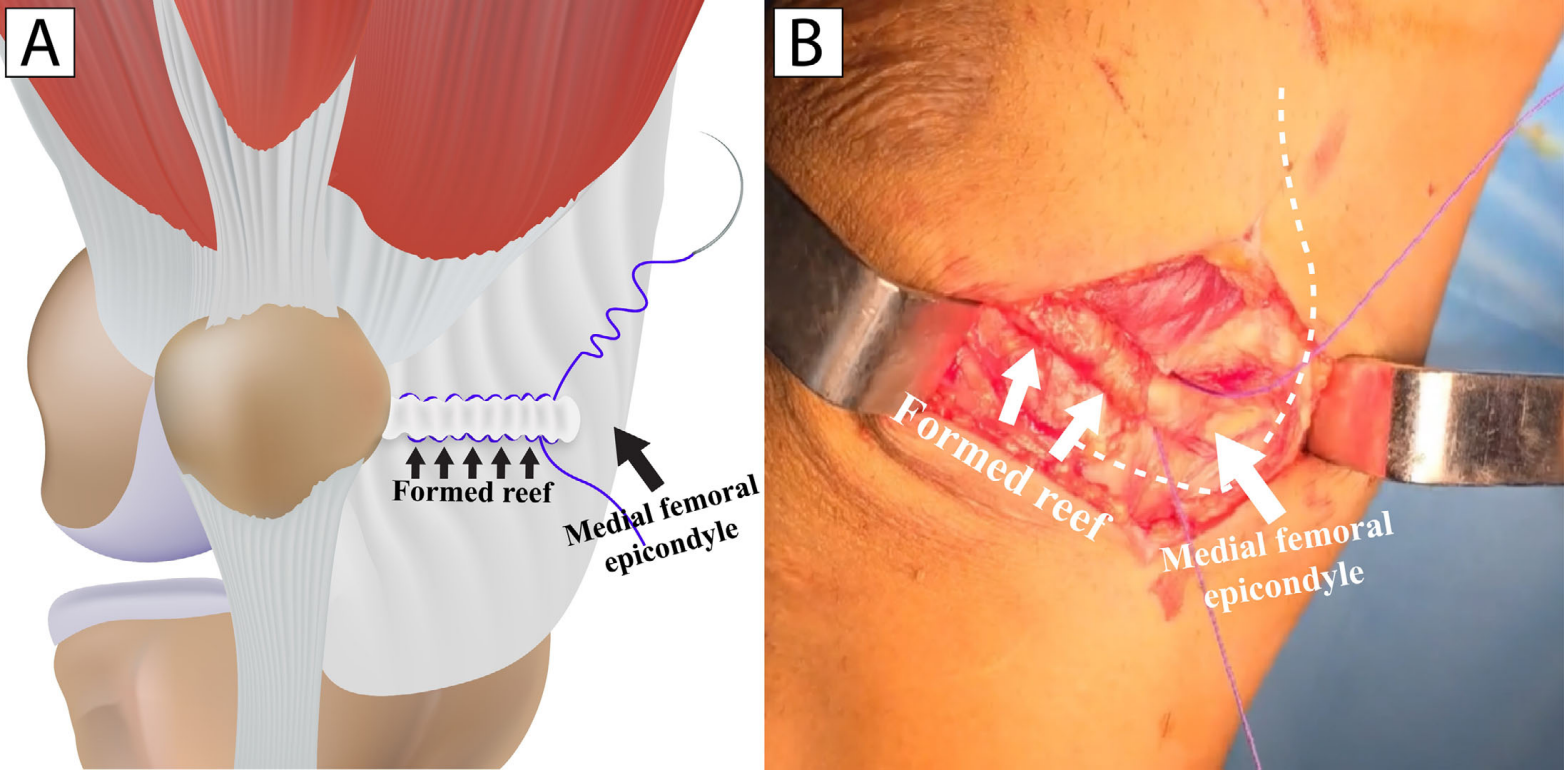
Formed reef. (A) Schematic view showing the completed reef formed by tightening the medial patellar retinaculum. (B) Corresponding intraoperative photograph of the formed reef. The 2 ends of the suturing thread are positioned on either side of the reef. The patient is positioned supine with the knee in midflexion; this is the right knee.

**FIGURE 5:**
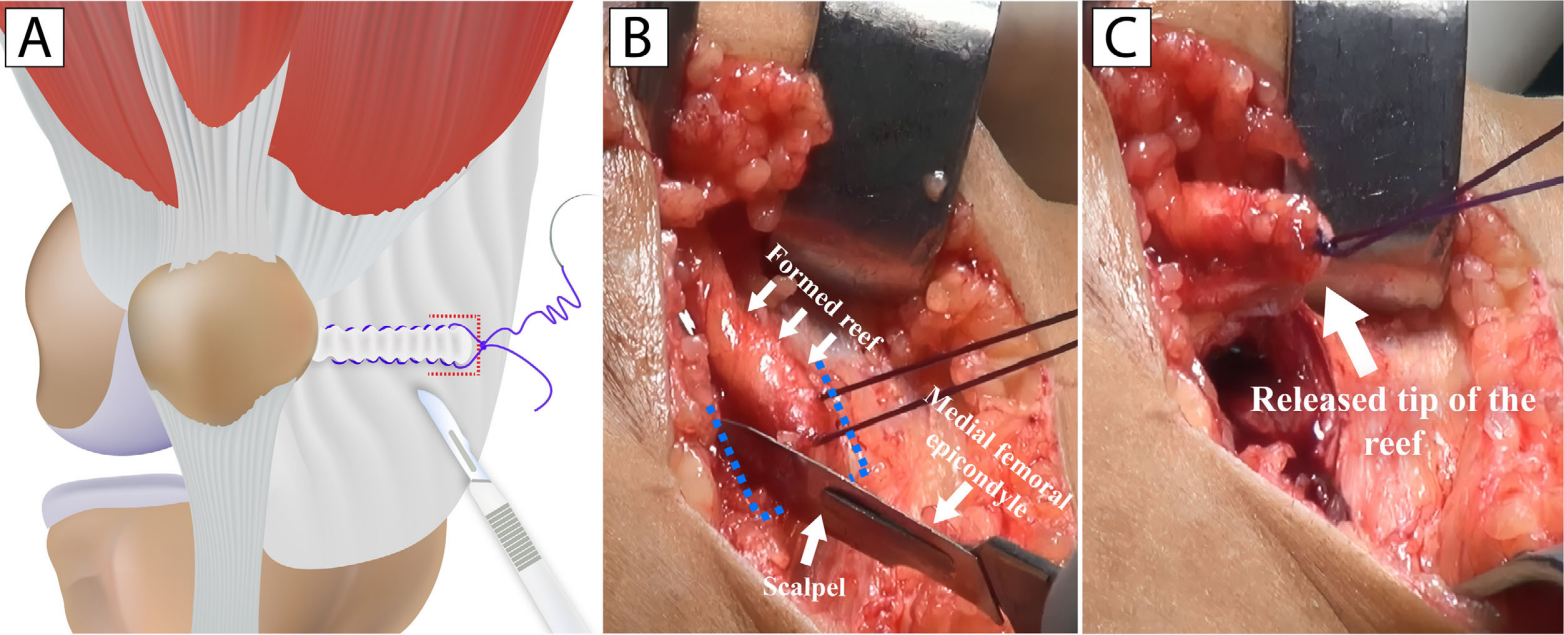
Releasing the tip of the reef. (A) Schematic illustration showing release of the reef tip. Three dashed red lines mark the proposed incision sites. The 2 ends of the sutures are knotted to balance tension and facilitate handling and mobilization of the reef after release. (B) Corresponding side view of the surgical field, showing the 3 incisions made on either side of the medial end of the tubed reef to release the tip. (C) Released reef tip after the incisions. The threads are knotted to each other. The patient is positioned supine with the knee in midflexion; this is the right knee.

### Femoral Pouch Development

A 2‐cm longitudinal incision is made over the medial epicondyle, precisely at the MPFL's femoral footprint, where the ligament naturally attaches (Figure 6). The femoral attachment site is identified using reliable anatomic landmarks, specifically the saddle‐shaped impression between the medial femoral epicondyle and the adductor tubercle, which corresponds to the native femoral footprint of the MPFL without the need for fluoroscopy or bone tunnels. The retinaculum is opened, and a subperiosteal fascial pouch is created over the medial epicondyle (Figure 7). Based on our experience, this method avoids the risk of fractures and enhances the biomechanical stability of the reconstruction compared with bone tunnel techniques. The released construct, which is now mobile and able to be pulled medially together with the patella for about 2 cm, is pulled underneath the created pouch and fixed with sufficient tension via sutures in the pouch as well as on either side of the construct (Figure 8, Table 1). The patella is checked for stability and then mobilized to check for optimal tension and to prevent overtensioning (Figure 9).

**FIGURE 6:**
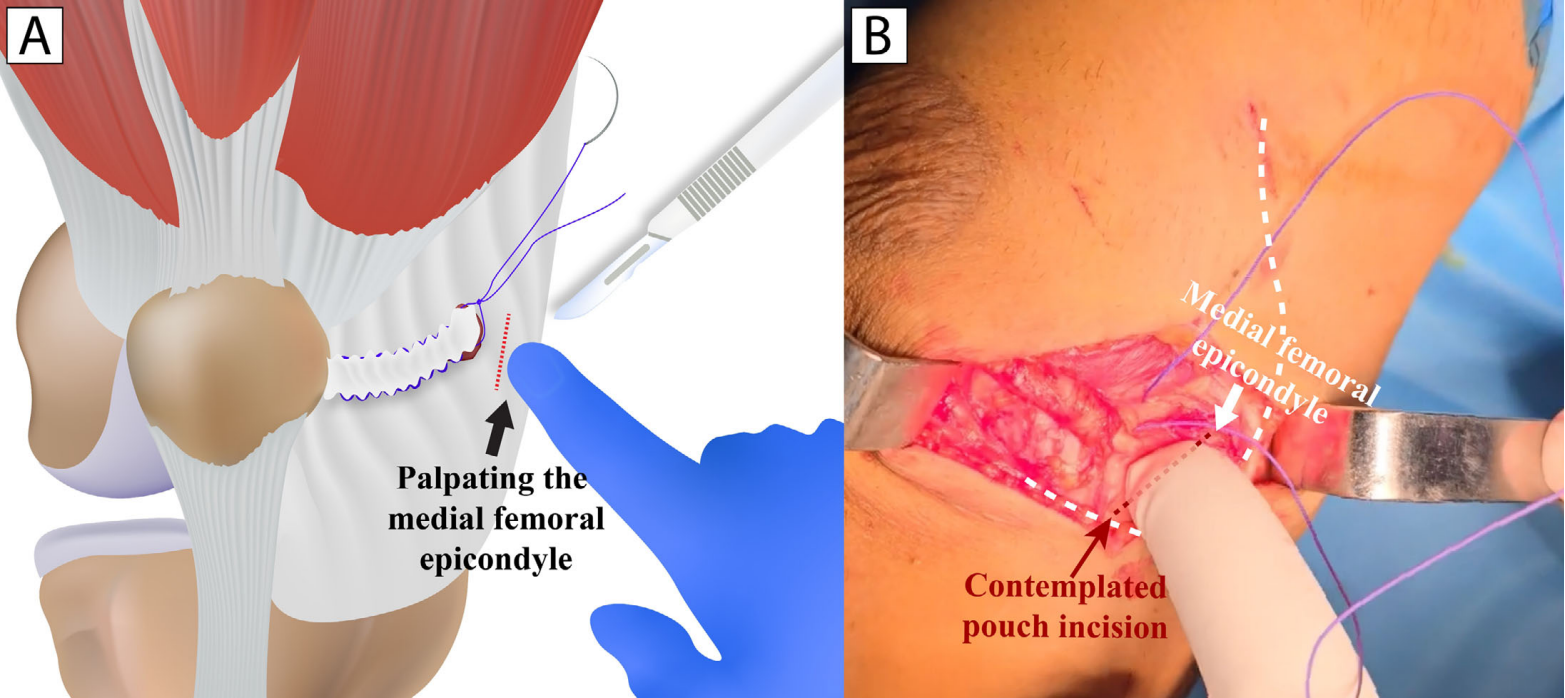
Palpating the medial femoral epicondyle to identify the incision site for pouch creation. (A) Schematic illustration showing the surgeon's finger palpating the medial femoral epicondyle. (B) Corresponding intraoperative photograph showing the same step. The patient is positioned supine with the knee in midflexion; this is the right knee.

**FIGURE 7:**
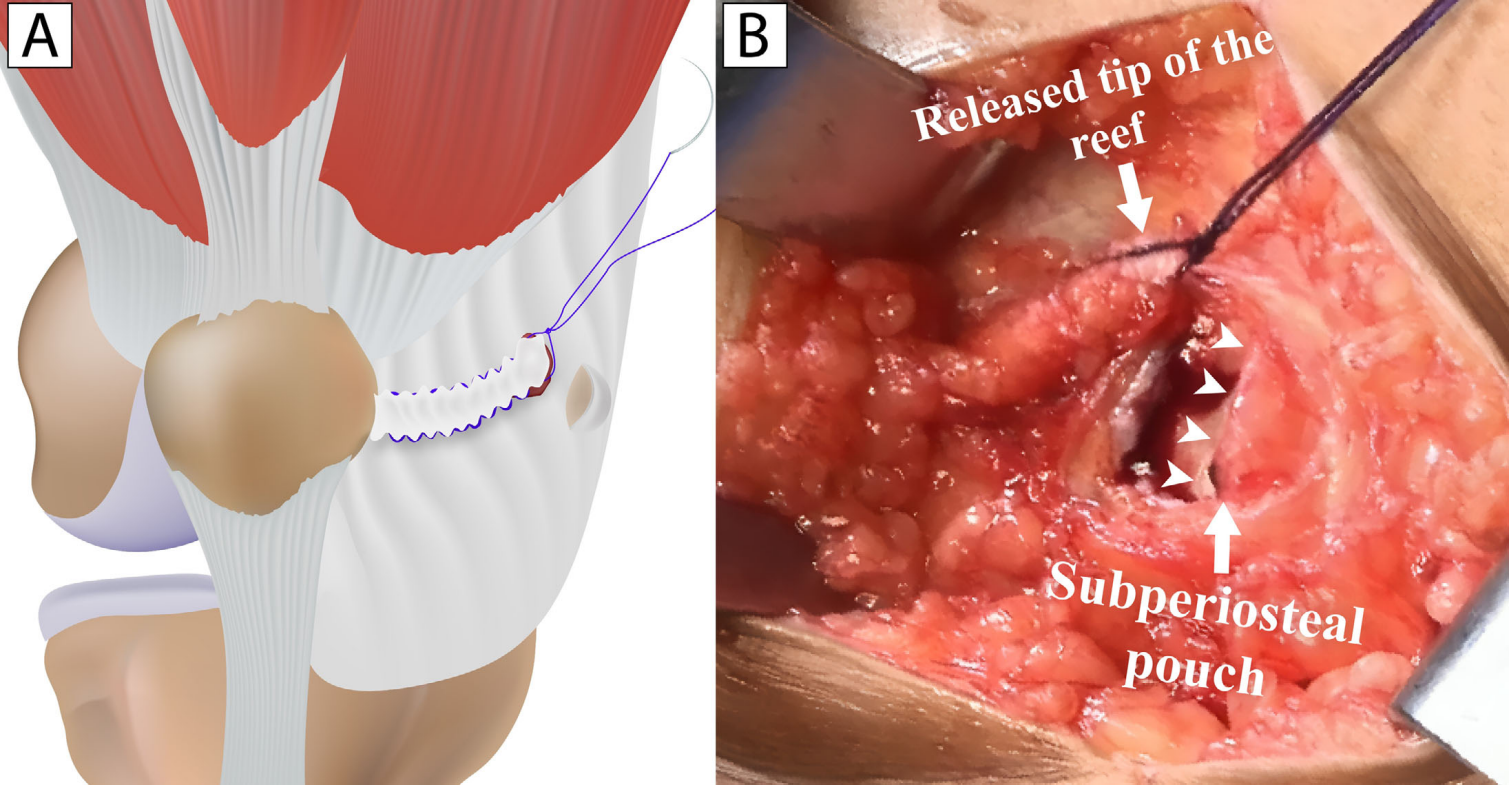
Postincisional view of the subperiosteal pouch created on the medial femoral epicondyle. (A) Schematic illustration showing the subperiosteal pouch. (B) Corresponding intraoperative photograph showing the subperiosteal pouch (white arrowheads). The patient is positioned supine with the knee in midflexion; this is the right knee.

**FIGURE 8:**
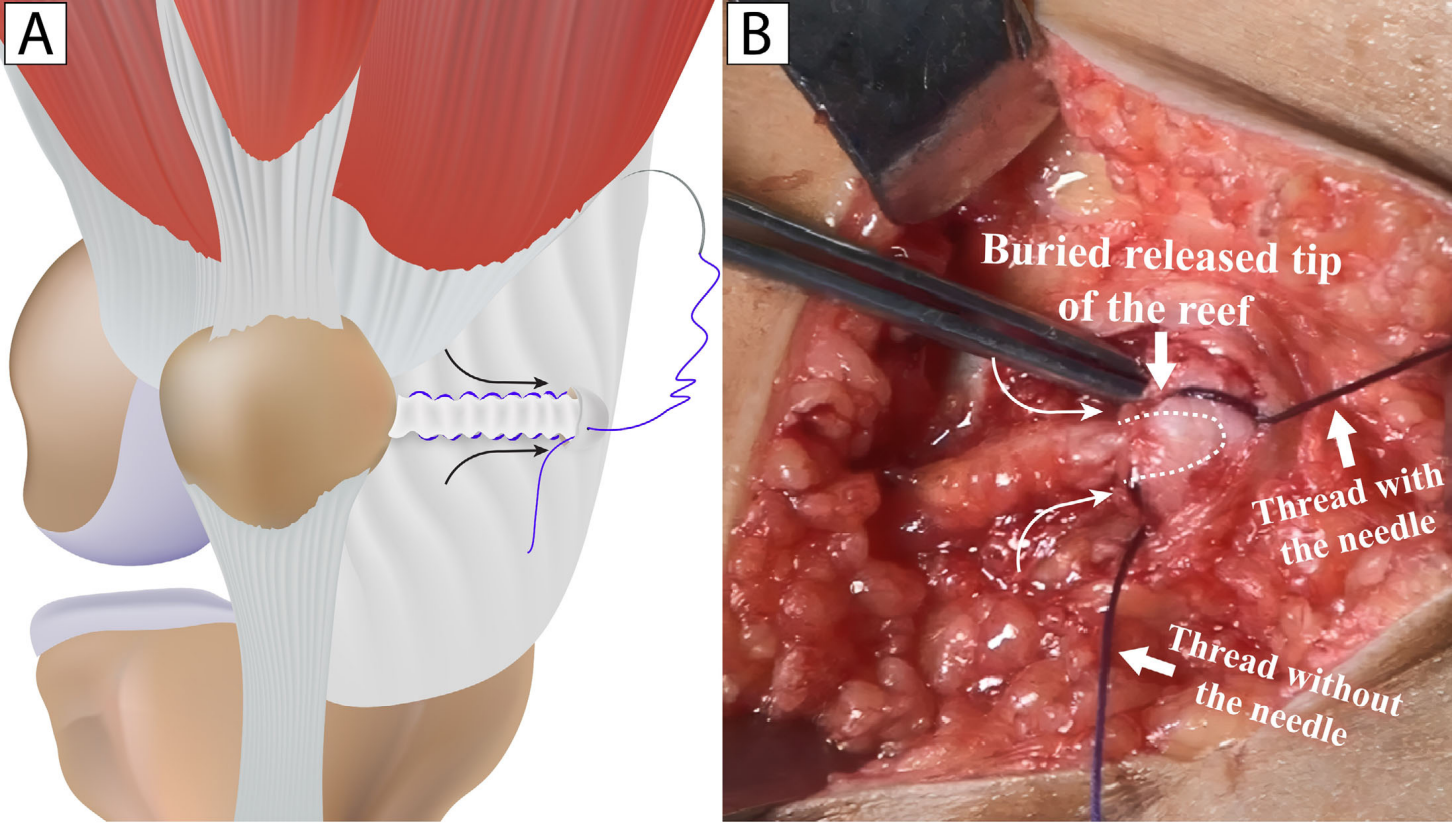
Burying the tip of the reef underneath the subperiosteal pouch. (A) Schematic illustration showing placement of the reef tip underneath the created subperiosteal pouch. Before positioning the tip of the reef, the suture end with the needle is passed under and then through the pouch wall, while the other suture end is left outside the pouch for the final surgical knot. (B) Corresponding intraoperative view of the positioned reef. The buried tip of the reef is marked by a dashed line. The patient is positioned supine with the knee in midflexion; this is the right knee.

**TABLE 1:** Pearls and Pitfalls of the Present Technique

**Pearls**
• Releasing the tubed construct at the femoral end by approximately 1 to 1.5 cm is sufficient.
• Fixing the ligament in the created pouch should be performed at midflexion with the patella engaged in the trochlea to prevent overtightening.
• In cases requiring femoral osteotomy, extending the medial incision allows both procedures to be conducted effectively.

**Pitfalls**
• Over‐ or undertightening of the reconstructed ligament may compromise the outcomes.
• In patients with patella alta, trochlear dysplasia, or malalignment, this procedure alone is insufficient and should be combined with corrective osteotomies.

**FIGURE 9:**
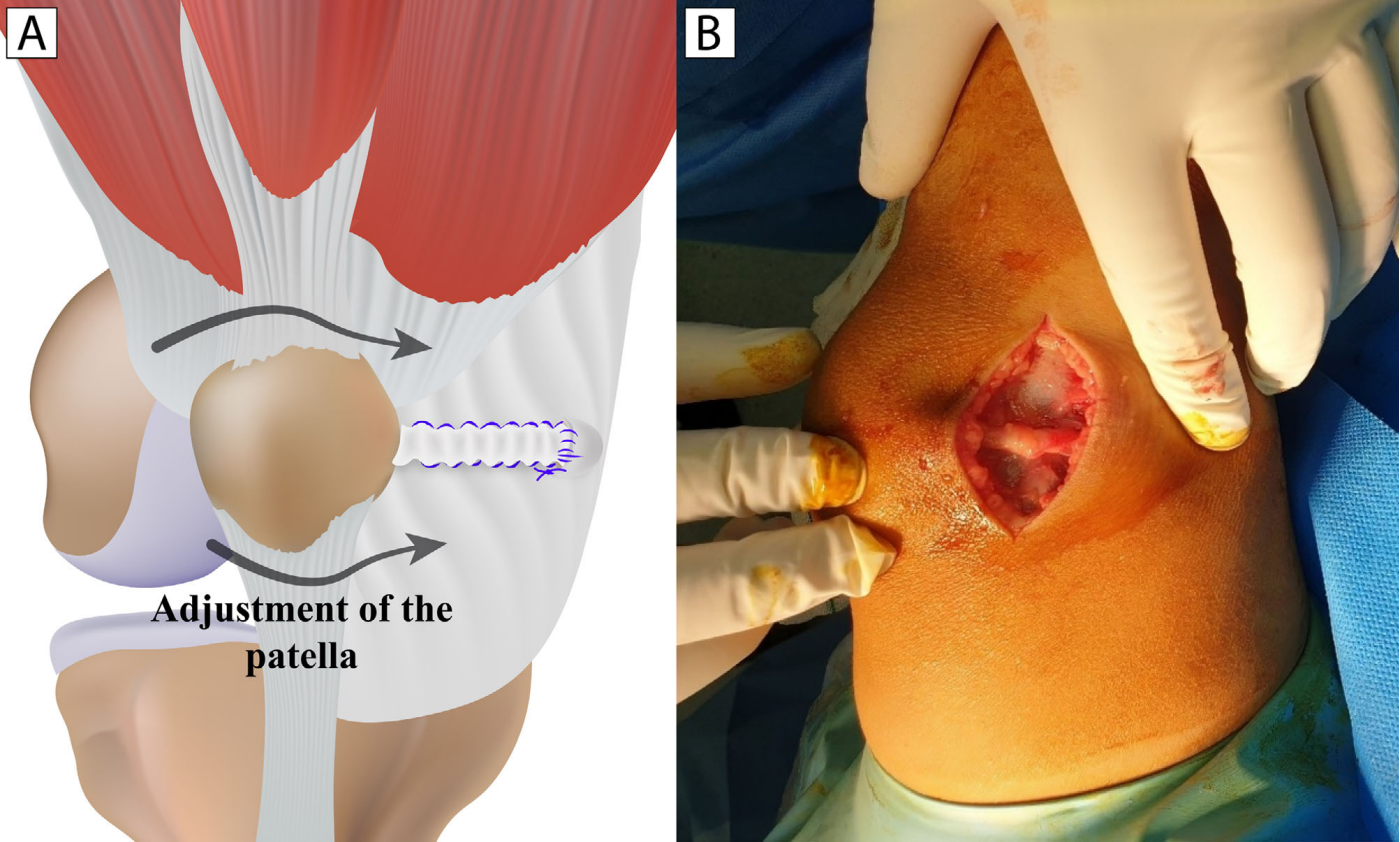
The reconstructed MPFL holding the patella in place. (A) Schematic illustration showing the reconstructed MPFL. (B) Corresponding postoperative photograph showing the reconstructed MPFL. Postoperatively, the surgeon checks patellar stability and optimal tension. The patient is positioned supine with the knee in semiflexion; this is the left knee. (MPFL, medial patellofemoral ligament.)

### Wound Closure and Postoperative Protocol

The skin is sutured; no drain is needed. A Jones bandage and knee immobilizer are applied. Early passive and active‐assisted range of motion is initiated on postoperative day 1 as tolerated, and partial weight‐bearing with crutches is allowed. At 1 week, dressings are removed, and formal physiotherapy begins with emphasis on quadriceps activation, edema control, and progression to full weight‐bearing over 4 weeks. Return to running is generally at 8 to 10 weeks, with sport‐specific training thereafter.

## DISCUSSION

A key aspect of orthopaedic ligament reconstruction surgeries is to use a technique that ensures excellent outcomes while minimizing complications. Despite this fundamental principle, the reconstruction of various ligaments across the body employs diverse techniques. This arises primarily because different techniques provide unique advantages but are accompanied by specific disadvantages. This variability leads surgeons to favor certain methods based on their training, expertise, and personal success rates. Nowhere is the lack of consensus more apparent than in MPFL reconstruction, which, although extensively researched, lacks a standardized technique.^8^, ^9^ In most MPFL reconstruction surgeries, surgeons create tunnels or sockets, through which the graft is passed using 1 or 2 tunnels drilled into the medial aspect of the patella.^10^ A large body of evidence has been dedicated to comparing the efficacy and safety of single‐ versus double‐bundle techniques.^11^ However, complications persist with both techniques, including postoperative apprehension and stiffness.^12^ The technique of creating bone tunnels in the patella and securing the graft with interference screws poses a risk of transverse patellar fracture, with the highest fracture rates reported when 4.5‐mm tunnels are drilled.^13^ In our technique, patellar fracture is expected to be nonexistent because we avoid any bone violation of the patella.

Another commonly practiced technique uses suture anchors for bony fixation to secure the grafted tendon to the patella. This method is comparatively less invasive and minimizes the risk of fracture. Although biomechanically sufficient for patellar fixation, it provides lower stiffness compared with interference screw fixation.^14^ Nonetheless, it still requires the use of implants.^15^ In our technique, in which the reefing is inspired by the medial retinaculum plication technique^16^ (customarily referred to as Campbell's technique^17^), we use the retinaculum, obviating the need for implants. As implants add a significant cost, this technique is particularly beneficial in resource‐limited settings because it overcomes the dependency on foreign‐sourced materials. Additionally, this technique provides a true MPFL reconstruction rather than a simple repair or imbrication because the medial patellar retinaculum is tubularized and reinforced to create a distinct, ligament‐like bundle that reproduces the native MPFL course and function. Although retinacular imbrication is performed concurrently, the construct is mobilized and biologically fixed at the anatomic femoral footprint, thereby establishing a new functional restraint that acts as a reconstructed MPFL rather than a mere retinacular plication.

Graft choice is another determining, yet often subjective, variable in MPFL reconstruction surgery. The graft source can range from autograft, allograft,^18^ or even synthetic.^19^ According to the systematic review by Aliberti et al., the gracilis tendon was the preferred autograft, whereas various soft‐tissue allografts, including semitendinosus‐gracilis, bone‐quadriceps tendon, tibialis anterior, and tensor fascia lata, were used.^20^ Furthermore, Migliorini et al. showed that using semitendinosus as the graft choice was associated with superior Kujala, Lysholm, and Tegner activity scores compared with the gracilis tendon in isolated MPFL reconstruction.^21^ Another recent study by Runer et al. showed that in isolated MPFL reconstruction, the quadriceps tendon autograft resulted in significantly lower donor‐site morbidity than the gracilis tendon autograft.^22^ In our technique, which uses the medial patellar retinaculum, we expect to see minimal to no donor‐site morbidity. Using the medial retinaculum as the graft appears to be easier and less invasive than other grafts.

In our technique, we create a subperiosteal pouch through a single incision. Fixing the tubed and reinforced construct to the periosteum over the medial epicondyle not only tightens the elongated medial retinaculum but also creates a strong, biological ligament that prevents the patella from dislocating. We expect it to be reproducible and accessible for less‐experienced surgeons. Like all surgical techniques, this method has potential limitations in some populations. One expected concern is that in patients with generalized hyperlaxity or connective tissue disorders, the retinaculum may not provide sufficient mechanical strength. In such cases, alternative grafts may be considered. Although further studies are needed to confirm long‐term outcomes, we present this reinforced horizontal retinacular reef technique as a viable, implant‐free option that addresses many limitations of existing methods (Table 2).

**TABLE 2:** Advantages and Disadvantages of the Proposed Technique

**Advantages**
• No need for implant use: the technique is biological and eliminates implant use, reducing associated costs and complications.
• No special instruments are required.
• Reproducibility: the technique can be easily performed by trained surgeons.
• No bone tunnels are required, eliminating the risk of patellar fracture.
• Prevents overtensioning.
• In addition to MPFL reconstruction, the retinaculum is tightened: provides dual benefits of anatomic MPFL reconstruction and retinaculum reinforcement.

**Disadvantages**
• Potential biomechanical insufficiency: the use of the medial retinaculum as the reconstructive substrate may provide less tensile strength compared with other grafts, theoretically leading to a higher risk of recurrent instability or graft failure. However, in our series, no failures have been observed to date. Further biomechanical studies are warranted to evaluate the tensile strength and long‐term durability of the retinacular construct relative to traditional graft options.
• Limited level of evidence: this technique currently relies on preliminary patient series data rather than robust trials, requiring longer‐term and further validation.
• Although theoretical concerns exist that the autologous retinacular construct may inherit the laxity seen in patients with generalized hyperlaxity or connective tissue disorders, our clinical experience indicates that these patients can still achieve satisfactory stability and functional outcomes.

MPFL, medial patellofemoral ligament.

## DISCLOSURES

The authors (H.M., S.H.M., A.V., M.H.E., M.S., R.G.) declare that they have no known competing financial interests or personal relationships that could have appeared to influence the work reported in this article.
